# Carbon Emission Risk and Governance

**DOI:** 10.1007/s13753-022-00411-8

**Published:** 2022-04-20

**Authors:** Lu Jiang, Xiaokang Hu, Gangfeng Zhang, Yanqiang Chen, Honglin Zhong, Peijun Shi

**Affiliations:** 1 Academy of Plateau Science and Sustainability, People’s Government of Qinghai Province and Beijing Normal University, Xining, 810016 China; 2 State Key Laboratory of Surface Processes and Resource Ecology, Beijing Normal University, Beijing, 100875 China; 3 Academy of Disaster Reduction and Emergency Management, Ministry of Emergency Management and Ministry of Education, Beijing Normal University, Beijing, 100875 China; 4 Faculty of Geographical Science, Beijing Normal University, Beijing, 100875 China; 5 Institute of Blue and Green Development, Weihai Institute of Interdisciplinary Research, Shandong University, Weihai, 264209 China

**Keywords:** Carbon emission risks, Carbon neutrality, Low-carbon economy, Risk governance, Vulnerability coefficient

## Abstract

Within the hazard and disaster risk research field, explicitly treating carbon emissions as a hazard remains rather nascent. Applying hazard and disaster risk research perspectives to seek new insights on integrated mitigation and adaptation approaches and policy measures is equally elusive. Since China’s pledge to achieve carbon neutrality by 2060, the “dual carbon” goals of carbon emission peaking and neutrality have stimulated nationwide attention, research, and policies and action plans. How to ensure that the transition pathways are on track and well-contextualized is one of the crucial challenges for policymakers and practitioners. This article examines the “risks” of missing the carbon neutrality goal at a regional scale in China, denoted as Carbon Emission Risk (CER). Carbon emissions (CE) as hazard, combined with the human socioeconomic system as exposure and human living environment, constitute the regional carbon emission environmental risk system. The “risks” of missing (or achieving) the carbon neutrality target for any region at any time, the article argues, is essentially determined by the ratio of CE to carbon absorption (CA, for uptake and removal). These variables are modified by a broadly defined “vulnerability coefficient” (Cv) that embodies both the potential for changes (decreasing CE and increasing CA), and the uncertainties of measuring CE and CA. Thus, the ratio of CE to CA is a measure of reality at any moment of time, whereas Cv indicates the overall propensity or capacity for moving the CE/CA ratio towards 1, that is, realizing carbon neutrality. Based on our calculation, CER at the provincial level in eastern China is higher than in western China. The article also calls for strengthening CER research and summarizes key measures for carbon emission risk governance.

## Introduction

Climate change due to a growing concentration of greenhouse gases in the atmosphere is a challenge for all of humanity, which has entered a “climate emergency” stage that requires urgent action. Achieving global net-zero emissions around the midcentury, which is required to cap global warming at 1.5 °C above preindustrial levels as called for in the 2015 Paris Agreement, was a central topic at the Glasgow UN Climate Change Conference (COP26), the latest in the series of Conference of the Parties Under the United Nations Framework Convention on Climate Change that began in 1992. While progress is being made with new declarations and higher ambitions, research suggests that even if all net-zero commitments and national climate pledges were fulfilled, warming would not be held to 1.5 °C above preindustrial levels. This +1.5 °C cap is a level projected to significantly mitigate, albeit still with increased climate-related risks to health, livelihood, food security, water supply, human security, and economic growth (IPCC 2018). At the same time, climate change is widespread, rapid, and intensifying, already affecting many weather and climate extremes in every region across the world, as identified in the recently released Sixth Assessment Report (AR6) of the Intergovernmental Panel on Climate Change (IPCC 2021; IPCC 2022).

In September 2020, China pledged to reach carbon neutrality by 2060, a progression from its present emission levels through a carbon emission peak in 2030 to carbon neutrality in 2060. These two goals have captured the Chinese nation’s imagination, galvanized carbon neutrality research efforts, and generated action plans tailored to the specific context of all regions and sectors. Fixed deadlines have helped to focus national effort. But achieving a proper balance between policy and practice in order to meet these deadlines will require flexibility, sacrifice, and persistence as well as hard decisions in policy and practice.

This challenge is by no means unique to China. Execution will not be easy: solving the net-zero equation cannot be divorced from decreasing carbon emissions (CEs) and increasing carbon absorption (CA). Doing so will require a careful balancing of the shorter-term risks of poorly prepared or uncoordinated action with the longer-term risks of insufficient or delayed action (McKinsey 2022). Moreover, environmental risks affecting sustainable global development that are attributed to global climate change should be simultaneously alleviated.

There are many ways to further frame this implementation challenge. Here we note three of them that motivate the current study. One has to do with the perception of, and in turn, the incentive for climate actions at regional and local scales. Another is the disjunction between mitigation and adaptation. The last issue is how to manage the “transition risks” that require careful balancing of economic development and climate security, as well as social impacts (that is, a just transition).

While awareness of the imperative for and urgency of climate actions is rapidly increasing, at the local scale, decision makers often perceive climate change as a global challenge that should be managed at the international and national levels. Thus, there is often a lack of sufficient incentive to translate the climate transition urgency into local development strategies and planning. One of the reasons for this perception is the fact that much of the targets and goals are derived from climate models and socioeconomic development pathways at the global and national scales, and in a top-down fashion. In other words, to reshape such a perception, a bottom-up approach is needed through which local actors can see a clear linkage between the required local actions and the national and global trajectories.

When it comes to local implementation of climate actions, what happens most often is the separation of mitigation and adaptation, each following separate strategies and action plans and aiming for different targets. The result is often the lack of policy coherence and low efficiency.

Lastly, measures of both mitigation and adaptation need to be contextualized. There are tremendous regional and local differences in terms of greenhouse gas emissions, impacts of climate change, as well as environmental and socioeconomic development conditions. For example, some areas have low emissions simply because of a low economic development level; others might be heavily dependent on fossil fuels or high carbon intensity industrial structures; still others might be rich with potential for sinking carbon due to specific land cover and land use patterns. What all places have in common is the need to manage the transition risks so that the journey towards low-carbon and sustainable development is stable, just, and highly adaptive to local conditions.

To address some of the above challenges, we believe that the notion of carbon neutrality—defined as a state of net-zero CO_2_ emissions, offers a useful framework, because it can be measured at all scales, it embodies both mitigation (cutting emissions) and adaptation (enhancing absorptions) measures and thus promotes integration. Thus carbon neutrality can provide progress-markers to innovate and adapt place-based transition pathways to local conditions.

Setting carbon neutrality as a desired system state and seeing deviations from such a state as risks, this article takes a hazard and disaster risk research perspective to seek new insights on integrated mitigation and adaptation approaches and policy measures. Following this introduction, Sect. 2 constructs a carbon neutrality-based risk index denoted as Carbon Emission Risk (CER) to examine and map the “risks” of missing the carbon neutrality goal at the provincial level in China. Centered on the core elements of CER, Sect. 3 outlines a broad CER research agenda, calls for a stronger contribution from the hazard and disaster risk research community towards a low-carbon and sustainable development agenda, especially at the local and regional scales, and focuses on implementation and application (UN 2015). Section 4 summarizes key measures for carbon emission risk governance.

## Carbon Emission Risk

Since the beginning of the Industrial Revolution, human activities are responsible for almost all of the increase of greenhouse gas emissions of which CO_2_ emissions make up the vast majority (NOAA 2021). Thus, carbon emissions are arguably the most common and urgent hazard facing the world today. How to manage this hazard is the core subject of extensive climate change mitigation research, as reflected in the recently released IPCC Sixth Assessment Report, and the concept of risk is central to all three AR6 Working Groups (IPCC 2022). Although the field of earth system science has included carbon peaking and realizing carbon neutrality tasks as the most important frontier of research, within the hazard and disaster risk research field, explicitly treating carbon emission as hazard and carbon absorption as resource is uncommon. Hazard and disaster risk research perspectives to seek new insights on integrated mitigation and adaptation approaches and policy measures remain in an early application stage.

### Carbon Emissions and Carbon Absorption

Carbon neutrality can only be achieved by balancing carbon emissions (CEs) with carbon absorptions (CAs) through carbon uptake and removal. Thus, the ratio between the two, rather than the absolute value of each, is the key concern in relation to achieving the carbon neutrality goal.

**Fig. 1:**
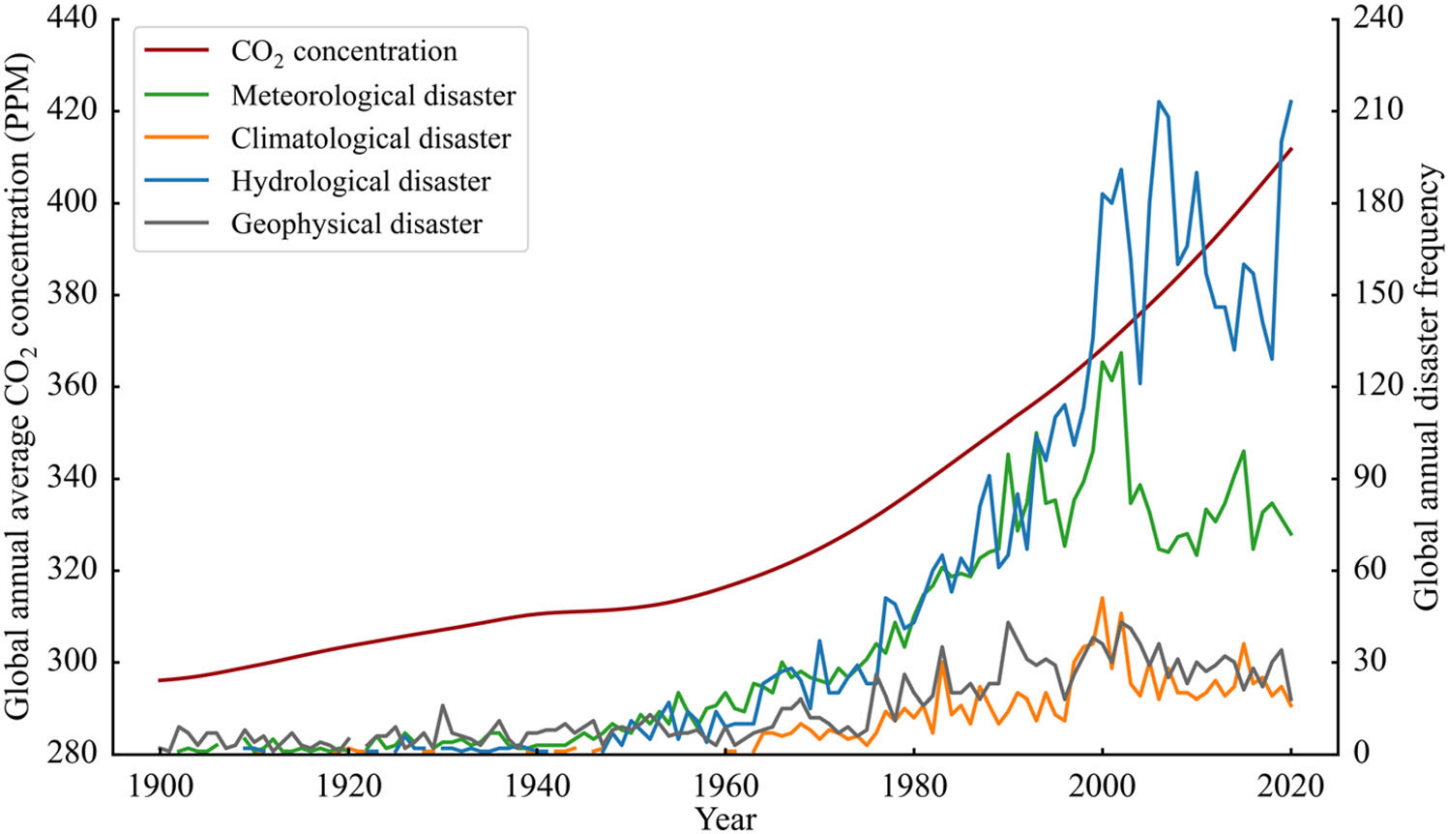
Correlation between extreme events caused by global climate change and the increasing CO_2_ concentration (SCP 2021; CRED 2022)

Human activities such as burning fossil fuels are the primary driver for the carbon emission hazard that causes not only global warming but also increases extreme meteorological, climatic, and hydrological events (Fig. 1) (Miner et al. 2021; Teng et al. 2021). In 2014, the Global Risk Report, released by the World Economic Forum in Davos, regarded global climate change caused by greenhouse gases such as CO_2_ as an important environmental risk factor (WEF 2014) and listed it as one of the top 10 global risks (WEF 2016).

Carbon emissions and carbon absorptions exhibit obvious regional differences in both time and space. Regional differences in CEs are closely related to regional economic and social development levels (Jiang, Xue, Ma, et al. 2020; Jiang, Xue, Xing, et al. 2020). The CEs in economically well-developed countries are higher than those in economically underdeveloped countries, the CEs in countries with a high degree of industrialization are higher than those in countries with a low degree of industrialization, and the CEs in countries with a high urbanization level are higher than those in countries with a low urbanization level (Wei et al. 2020).

Regional differences in CA are related to vegetation coverage, especially regional differences in forest cover. Thus, CA is associated with the climate zone, landform undulations, distance to the sea, and urbanization, industrialization, and agriculturalization levels of a region (Plaza et al. 2019). In general, forestlands exhibit the highest CA levels, followed by grasslands, whereas deserts exhibit the lowest CA levels. Areas with high urbanization rates contain less CA, and industrial and mining areas contain reduced CA. Except for desert oases, agricultural areas contain fewer CA than do natural vegetation areas within the same region (Huang et al. 2007). In recent years, scholars have paid more attention to ocean and cryosphere CA processes. Although the carbon storage amounts in these areas can be notable, given the same area, the CA level is much lower in these areas than that of forestlands (Roobaert et al. 2019; Ran et al. 2021).

Not only do significant regional differences occur in CA and CE, but also significant uncertainties and changes take place in these variables in both time and space. From a spatial perspective, CA is influenced by regional development policies. For example, the regional development policies implemented in China during different periods (Five-Year Plans, and so on) are associated with different CE levels (NPC and CPPC 2021). Since the reform and opening up in the late 1970s, the Chinese government has strengthened the economic development of coastal areas, which have become the fastest-growing region in China in terms of CE. From the perspective of time, dynamic changes in human social and economic activities have caused uncertainty in CA levels.

Studies have found that due to the impact of the Covid-19 pandemic, global CEs have been reduced (Le Quéré et al. 2020). When major celebrations were held in Beijing, China, high-emission factories in the surrounding areas were temporarily shut down, and CEs were significantly reduced. Because surface vegetation is affected by climate change, the CA capacity of surface vegetation exhibits obvious seasonal and interannual variabilities. In general, in months and years with higher precipitation and temperature levels, the CA capacity of terrestrial ecosystems is higher. Conversely, in months and years with lower precipitation and temperature levels, the CA capacity of terrestrial ecosystems is lower (Choat et al. 2012).

Research results in recent years have revealed that the CA of the oceans and cryosphere has also exhibited obvious uncertainty under global warming (Ran et al. 2021). Regarding oceans, global warming and temperature rise have increased CA. In terms of the cryosphere, the CA of permafrost has decreased, while the CA in seasonally frozen soil regions fluctuates. The CA in alpine desert areas is decreasing, the CA of alpine grasslands and meadows is increasing, and the CA in alpine forest areas is decreasing. These phenomena are related to an increase in soil CEs (Hong et al. 2020).

### Carbon Emission Risk Calculation

The above analyses indicate that there exist obvious temporal and spatial variabilities and uncertainties in CE and CA, as does the CE/CA ratio—an indication of carbon neutrality. To situate those concepts into coupled human environment systems and apply the regional disaster system theory (Shi 1991, 2018), this section makes a first attempt to construct an index that can offer a measure of differing risks of deviating from carbon neutrality across regions (or sectors and communities).

**Fig. 2:**
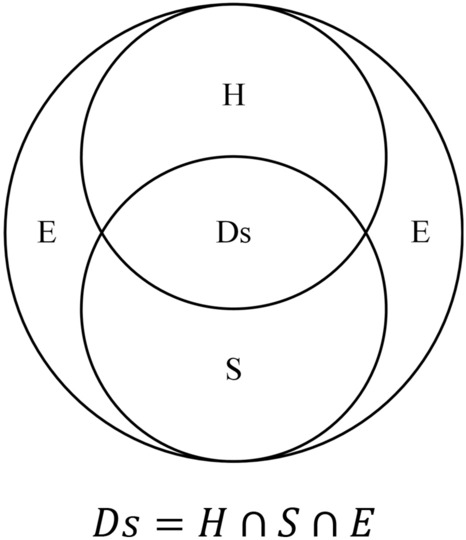
Carbon emission environmental risk system (Shi et al. 2015)

From the perspective of regional disaster systems and in the context of climate change, carbon emission (CE) constitutes a hazard factor (H) that causes climate warming and change. These changes, in turn, bring a wide range of increasing climate-related risks for ecosystems, biodiversity, and human systems that are interconnected. The coupled human-environment system (including human society, ecosystem dynamics, biodiversity, and so on) forms both the disaster-generating environment (E), and the elements of exposure (S) (Fig. 2). Here, the H encompasses both the primary carbon emission (or more accurately, the greenhouse gas emission) hazard factor that causes climate warming and a wide range of secondary climate-related hazards (for example, floods, droughts, storms, and heatwaves) that intensify as the result of climate change. Ds is the resultant climate-related disaster risks. Obviously, reducing the Ds requires managing all three contributing factors, that is, hazard, exposure, and vulnerability that are embedded in the disaster-generating environment (E).

For much of the natural-hazard focused research tradition, it was generally viewed that we have limited scope to alter or manipulate the hazard factors, although understanding of hazard formation processes and dynamics is essential for better forecasting and early warning. Clearly, this is not the case with the human activity-induced carbon emission hazard, which could by itself be viewed through a “risk” lens, that is, carbon emission risk. In other words, we argue that human activity-induced carbon emission has dual characteristics—it is a “hazard” in the context of causing climate warming, at the same time, it can also be seen as a “risk” in the context of maintaining a carbon neutral system state.

It is in this context that we define the carbon emission risk (CER) of a region (or a place, sector, company) as the risk of deviating from carbon neutrality. Because the key factor for measuring carbon neutrality is not the absolute amount of CE and CA per se, but rather the ratio between the two. Thus, in an ideal situation in which all needed information is available and accurate for calculating CE and CA, an index of CER can simply be expressed as CE/CA. In which case, when CER is 1, there is an exact balancing of the carbon emissions with carbon absorptions. Thus, CER = 1 can be interpreted as a carbon neutrality-based carbon emission risk benchmark. When CER is greater than 1, it indicates that the system (be that regional or sectoral) has a higher carbon emission risk, that is, it contributes to the growth of carbon emission globally. When CER is less than 1, it indicates a surplus of carbon absorption capacity (as resources to balance off carbon emissions), thus a lower carbon emission risk exists.

Reality is far from the “ideal”, however, and we can never have perfect information for CE and CA. The difficulties of capturing accurately measured CE and CA levels are many. Following the discussions in Sect. 2.1, we can broadly group these issues under two main aspects. One is the dynamic nature of both CE and CA, and the other is the uncertainties associated with measuring CE and CA at any point of time. To accommodate these difficulties, we modify the CE/CA ratio with a broadly defined “vulnerability coefficient” (Cv) that embodies both the potential for changes (that is, decreasing CE and increasing CA), as well as the uncertainties of measuring CE and CA, hence the construct of a carbon emission risk index (CER) as Eq. 1.

1 $$\begin{array}{c}{\text{CER}}={\text{CE}}/{\text{CA}}\times{\text{Cv}}.\end{array}$$

In Eq. 1, CER is a carbon emission risk index that measures the risks of missing (or achieving) the carbon neutrality target for any region at any time. CE is the carbon emissions per unit of time and per unit of area (g C/(m^2^ a)), CA is the carbon absorption per unit of time and per unit of area (g C/(m^2^ a)), and Cv is a vulnerability coefficient that modifies the CE/CA ratio. The CE/CA ratio is a measure of reality at any moment of time, whereas Cv indicates the overall propensity or capacity for moving the CE/CA ratio towards 1, that is, realizing carbon neutrality.

The vulnerability coefficient (Cv) is the least clearly defined parameter in this construct and it requires further study to clarify and improve. Considering the temporal and spatial characteristics of human socioeconomic activities, the Cv varies partially depending on the level of progress of low-CE industrial technology and high-CA biotechnology.

**Fig. 3:**
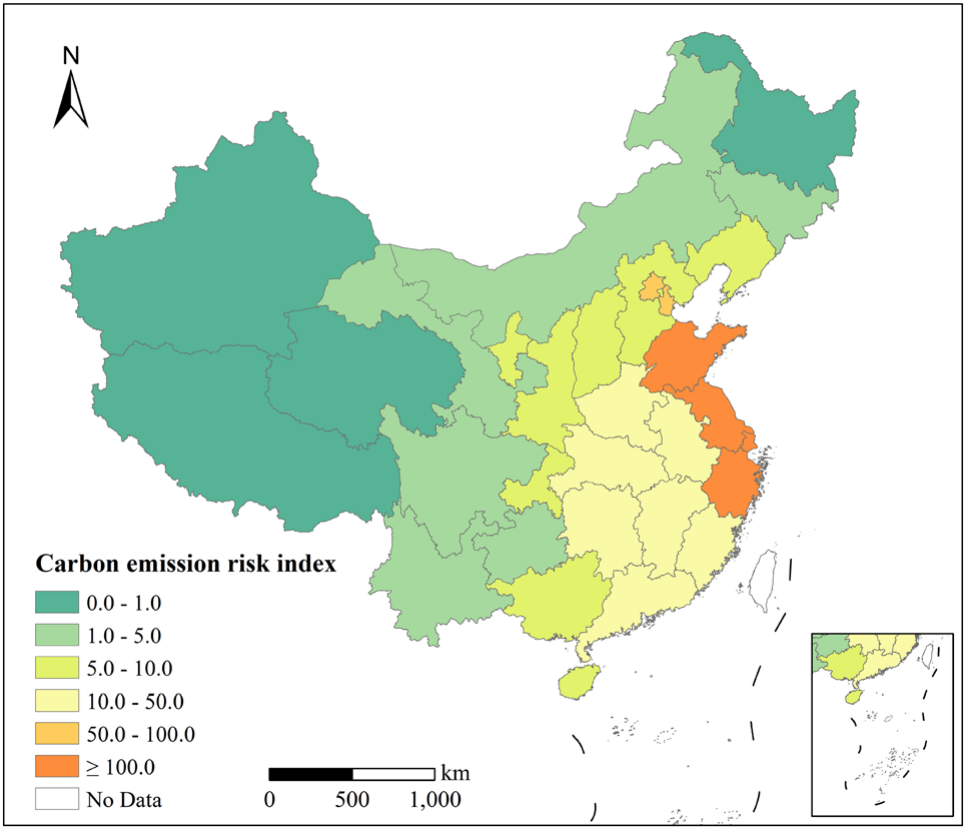
Carbon emission risk distribution at the provincial level in China (based on carbon absorption data from Peking University, Lu et al. 2018)

In this article, to test this initial CER index constructed to map the carbon emission risks at the provincial level in China (results shown in Fig. 3), we simply used per capita GDP as a surrogate for Cv based on the following considerations. The human socioeconomic system not only impacts CE but also impacts CA. In general, at the current state of development, in regions with a high socioeconomic development level, the CEs attributed to human activities usually exceed their CA capacity, which relatively amplifies the CER. In economically underdeveloped regions, human activity-induced CEs are usually lower than their CA, which relatively mitigates the CER. The CER at the provincial level in eastern China is higher than CERs in western China (Fig. 3). Clearly, there are many shortcomings with this gross simplification.

The Cv value should be calculated for specific regions or sectors, companies, and communities. The Cv value involves the energy structure, industrial structure, land use structure, natural ecosystem service value, and so on. It also includes changes in regional adaptation and mitigation measures resulting from technological progress. In actual calculations, vulnerability can be measured by the per capita GDP. Higher per capita GDP means greater exposure, but higher GDP may also indicate a higher level of progress on low-CE industrial technology and high-CA biotechnology. Based on the data from global generalized climatological disaster loss (CRED 2022), global average annual CO_2_ concentration (SCP 2021), and global GDP per capita (World Bank 2021), a multiple linear regression model of global generalized climatological disaster loss (*y*), global average annual CO_2_ concentration (*x*_1_), and global GDP per capita (*x*_2_) was constructed as *y* = 2.288*x*_1_ − 0.012*x*_2_ − 678.340, with a *R*^2^ of 0.582 and the *P* value is less than 0.001.

**Fig. 4:**
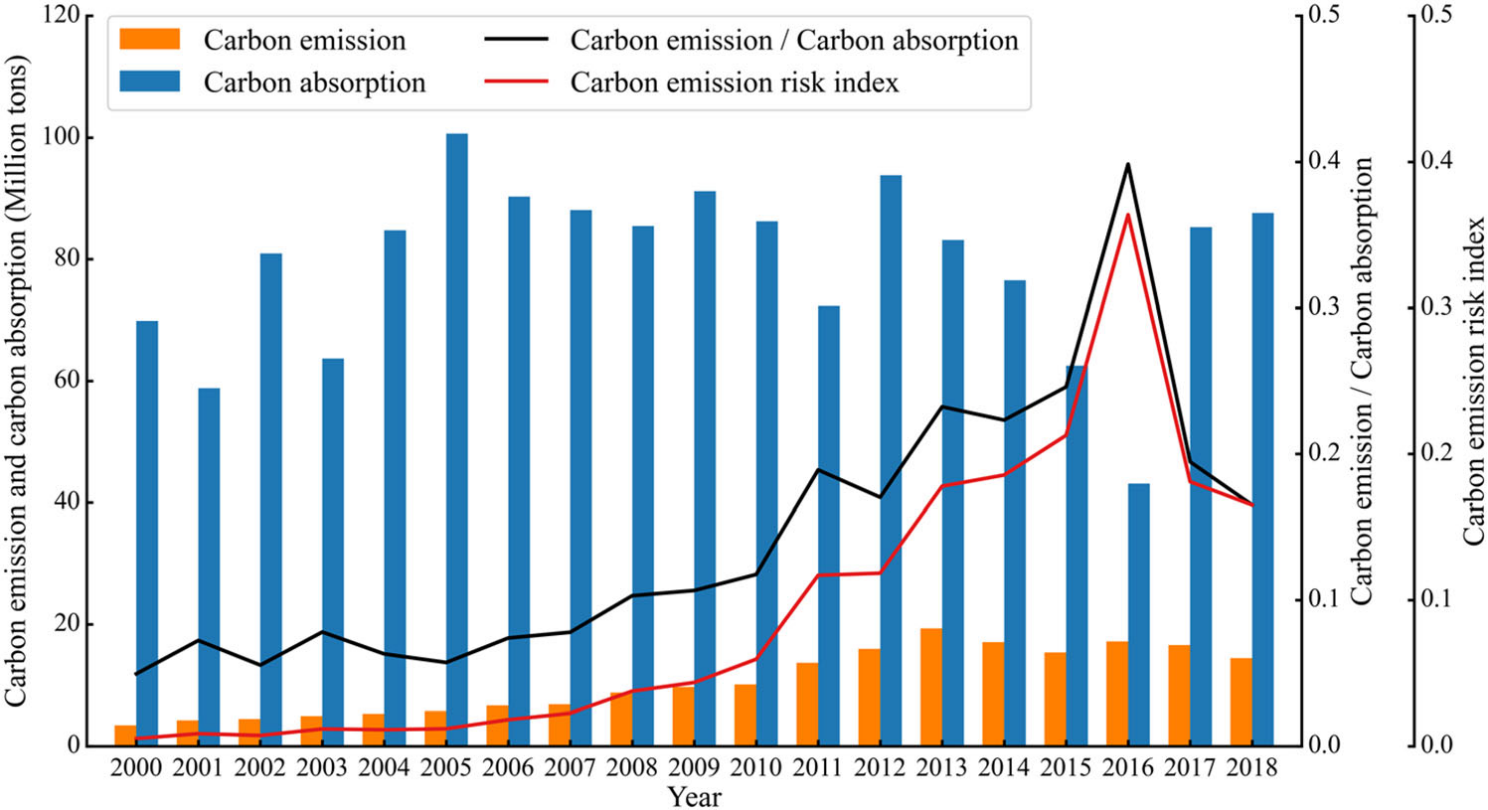
Results of the simulated carbon emission risk in Qinghai Province, China

In addition to spatial mapping, the CER construct can also be applied for longitudinal analysis. As a testing and illustrative case, we simulated the CER in Qinghai Province from 2000 to 2018 (Fig. 4), and we found that the CER exhibited an increasing but fluctuating trend. This phenomenon may be related to the reduction in CA caused by the degradation of frozen soil in the Qinghai-Tibetan Plateau under the background of global warming. The anomaly in 2016 reflects the impact of climatic drought conditions on ecosystem CA, which also indicates that under global climate change, CER amplification caused by the superposition of extreme climate events and climate change trends should be given adequate attention.

## Carbon Emission Risk Research

Based on the assessment of regional CER, CER research can provide important support to the timetable calculation of the regional carbon peak and carbon neutrality goals. This represents an important way to better understand the temporal and spatial differences and dynamic changes in CE, CA, and progresses towards carbon neutrality, and should be an important part of environmental risk research about global climate changes. CER research also can develop tools and methods to evaluate the feasibility of achieving carbon neutrality at the global, national, and regional scales. Based on the fundamental theories and methods of disaster risk science, this study established a basic framework for CER research.

### Linkages Between the Carbon Emission Risk and Global Climate Change Research

**Fig. 5:**
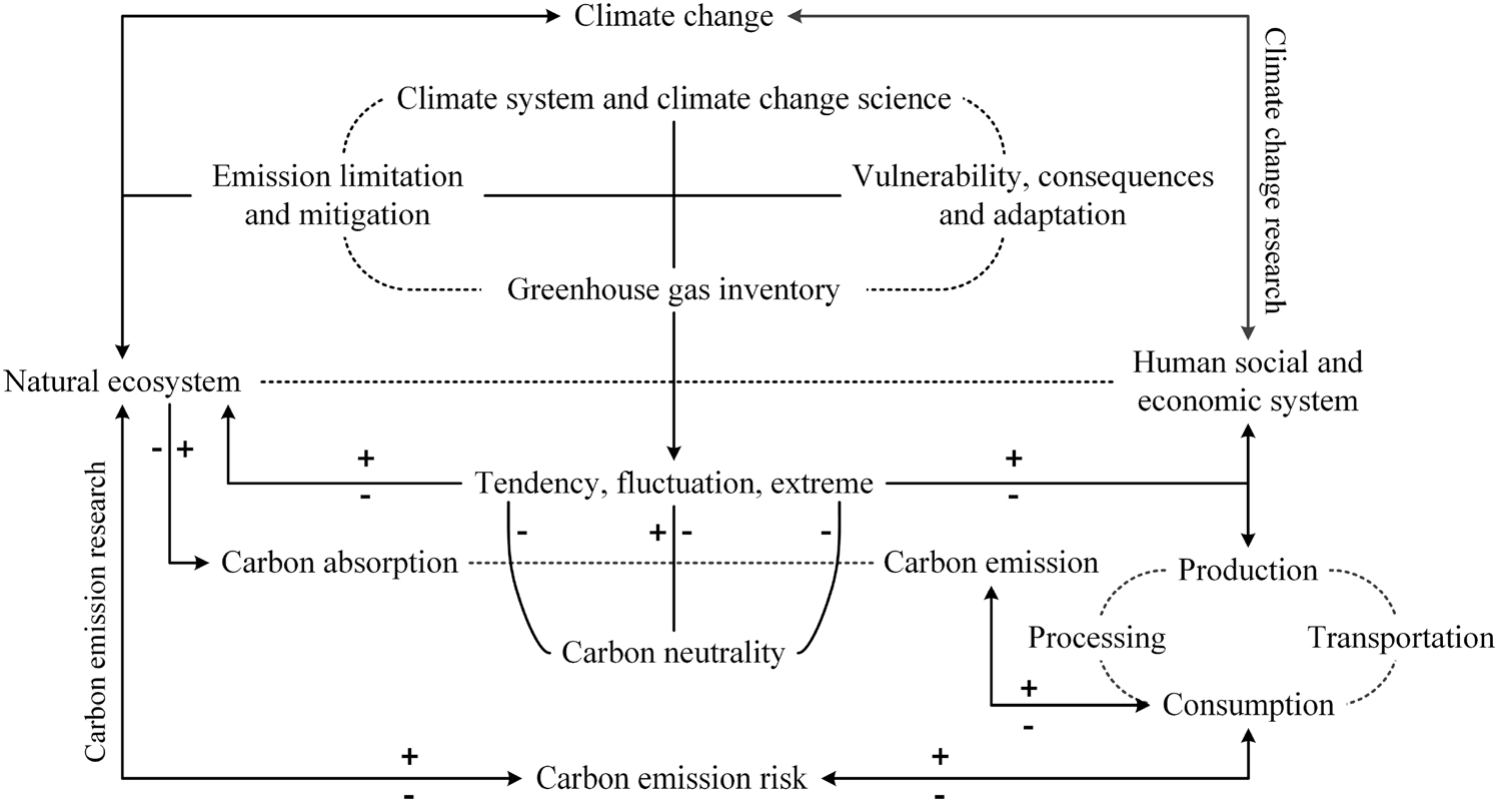
Diagram of the association between the carbon emission risk and global climate change research

There is a close relationship between the CER and global climate change research; CER research covers all aspects of the IPCC work (Fig. 5). First, global climate change is caused by greenhouse gases with CO_2_ at the core. This suggests that CER research should comprise an important part of global climate change research, and global climate change science represents the scientific basis of CER research. Second, the root of the CER entails human socioeconomic activities. In particular, without CEs due to human socioeconomic activities, there exists no CER, which suggests that the CER is an important research topic in geography, which has the study of human–environment relationships and regional systems at its core. Third, CER research should comprehensively capture the irreplaceable role of natural ecosystems in CA, which was emphasized during the Glasgow climate conference. To achieve global carbon neutrality, we must clarify the decisive role of vegetation protection, restoration, and construction in understanding the CER. Fourth, CER research must be incorporated into the overall framework of climate change risk research, and it must consider the trend of the climate change risk based on previous studies on extreme risks and climate fluctuation risks. Notably, CER encompasses the systematic risk caused by climate trends and the very high risk of CEs accumulation. Fifth, it appears clear that the CER is synergistically related to climate change, natural ecosystem dynamics, and human socioeconomic system research, which explains the multi- and interdisciplinary characteristics of CER research.

### Framework of Carbon Emission Risk Research

**Fig. 6:**
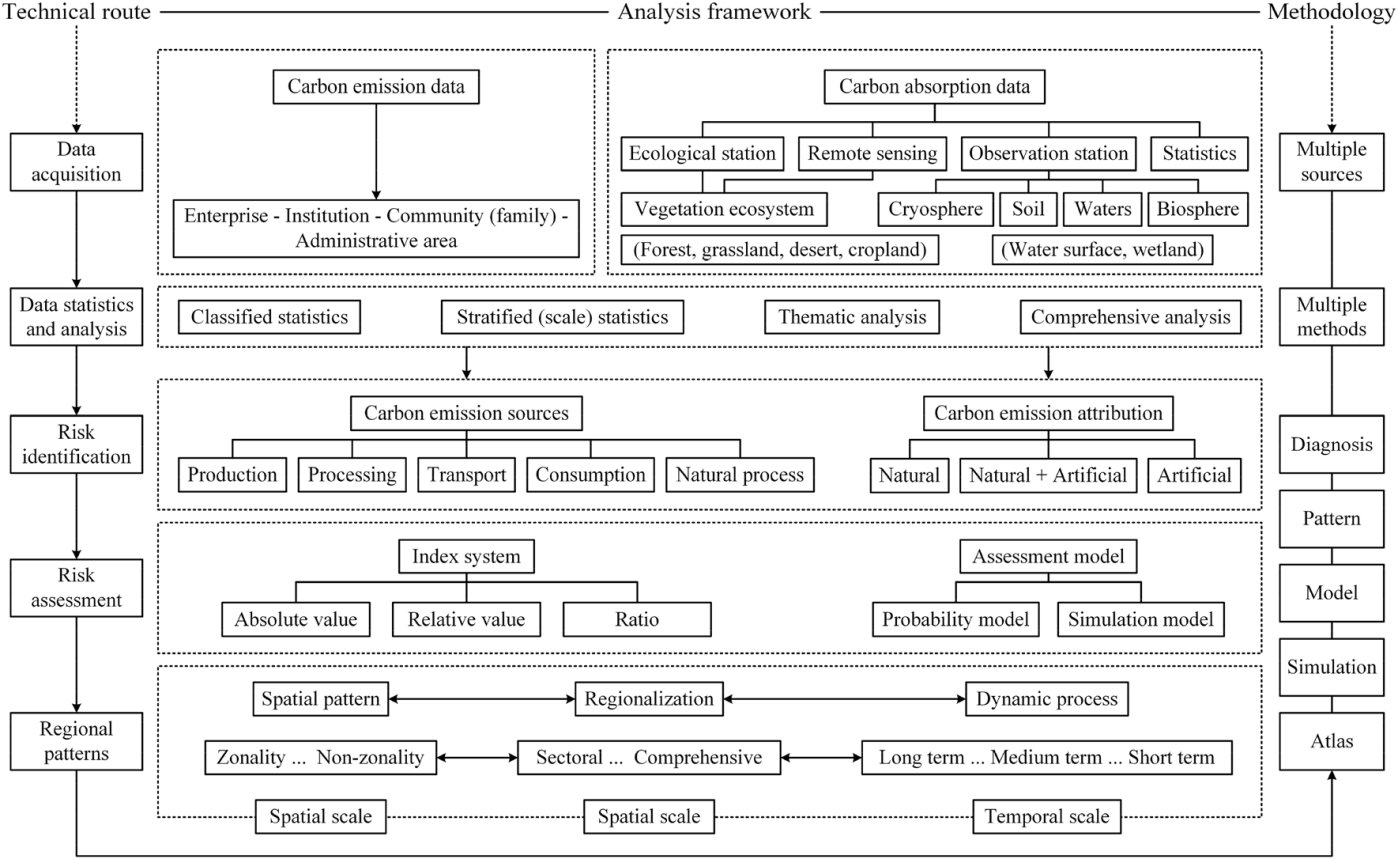
Carbon emission risk research framework (technology roadmap)

The preceding analysis indicates that CER research should learn from research methods and be based on the support of climate change science, geography, ecology, disaster risk science, and system science. Also it is necessary to establish a methodology and technical route considering multi- and interdisciplinary research (Fig. 6).

As shown in Fig. 6, the CER research framework includes three parts: the technical route, analysis framework, and methodology. Among these parts, the technical route conforms to that found in disaster risk science research, including data acquisition, statistical analysis, risk identification, risk assessment, and regional patterns. The analysis framework corresponds to the technical route, including classification, stratification, thematic and comprehensive analyses of CE and CA data, source and attribution analysis, assessment index and model, spatial pattern, dynamic processes, and regionalization. The methodology involves multiple approaches and methods, including diagnosis, pattern analysis, modeling and simulation, and atlas compilation. The technical route follows the fundamental paradigm of disaster risk science research. The analysis framework draws lessons from the integrated and regional analysis methods common in geography and the macroscale system analysis approach of system science. The methodology mainly encompasses diagnosis techniques and models of climate science, and methods of geography, such as map visualization.

CER research focuses on the “emission limitation and mitigation” concerned by IPCC Working Group III, that is, reducing carbon emissions by adjusting the regional economic structure and development pathways. At the same time, it also pays attention to the “adaptation” concerns that are the focus of IPCC Working Group II, that is, to increase CA by adjusting regional land use structure and pattern to adapt to climate change. In CER research, the risk of carbon emissions is the ratio of regional CE to CA, modified by the dual uncertainty of CE and CA levels. The research framework presented in Fig. 6 can be used to simulate CE and CA and to estimate the possibility or time of realizing CE/CA = 1 in the future. These estimates can provide an important scientific and technological support to the timetable calculation of the regional carbon peak and carbon neutrality goals.

Different assessment units can be selected in the CER calculation, such as families and communities, enterprises and institutions, and different levels of administrative units. The CER calculation can also target network units (such as kilometer-scale grids). Different time periods also can be selected in the CER calculation, such as day, month, quarter, year, decade, and century. The spatial unit and period of CER assessment mainly depend on the spatiotemporal resolution and accuracy of the obtained data. With improvement in various observation methods, big data, and artificial intelligence, as well as the rapid advancement in remote sensing Earth observation technology, and the densification of surface observation stations and ecosystem observation positioning stations, the accuracy and spatiotemporal resolution of CER assessment can be greatly improved.

## Carbon Emission Risk Governance

**Fig. 7:**
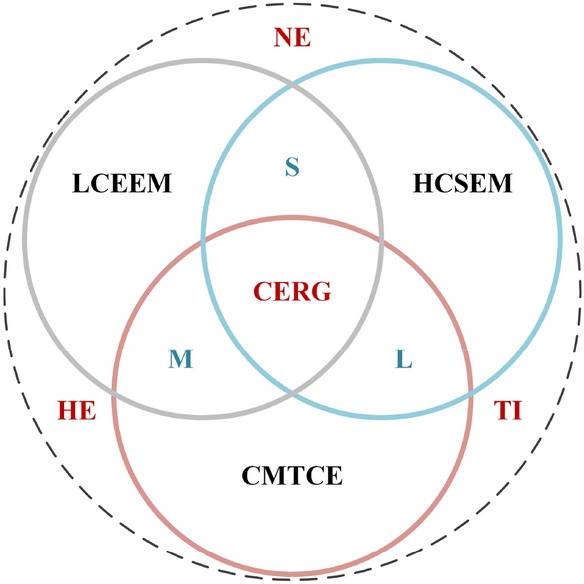
Regional carbon emission risk governance model. *LCEEM* low-carbon emission economic model, *HCSEM* high-carbon sink economic model, *CMTCE* complementary mode of traditional and clean energy, *CERG* carbon emission risk governance, *NE* the natural environment, *HE* the human environment, *TI* technological innovation, *S* system, *M* mechanism, *L* legislation

To achieve regional, national, and global sustainable development goals, the United Nations has developed a series of action plans and frameworks to address global climate change risk, disaster risk, and poverty reduction (UNISDR 2015; IPCC 2022). Among them, the realization of carbon peaking and carbon neutrality goals is regarded as a high-priority, and worldwide strategic action in response to global climate change and to achieve disaster risk reduction are considered essential. From the perspective of CER research, we propose integrated countermeasures based on the two-way force of CER governance and coordinated responses among regions (Fig. 7).

### Establishing a Low-Carbon Emission Economic Model—Comprehensive Promotion of a Low-Carbon Economy

At the beginning of this century, Britain launched a low-carbon economic model to address global change (DBIS 2015), and this model has attracted extensive attention globally. A low-carbon economy emphasizes economic structures and land use patterns with low CEs; focuses on reducing the CE intensity per unit of product output in the fields of production, processing, transportation, and consumption; stresses the 3R (reducing, recycling, and reusing) resource development model and utilization concept of resource conservation and environmental friendliness; develops a recycling economy; and constructs a low-carbon economic model. Under the guidance of this low-carbon strategic model, many countries have adopted low-carbon technologies in many fields to link CE reduction with environmental pollution prevention and control, especially the prevention and control of atmospheric environmental pollution. China’s major environmental protection policies, including the Waging a Blue-Sky Defense War initiative (SCPRC 2016), exemplify these principles and objectives. Through a series of measures, such as independent innovation, many countries have defined carbon peak targets, carbon neutralization roadmaps, and timetables.

According to the information acquired from the Glasgow climate summit, most developed countries have announced that they would reach a carbon peak during the 2025−2030 period (UK Government 2021). Achieving the goals of peaking carbon emissions and subsequent transition to carbon neutrality is one of China’s major strategies, defined after careful consideration. To this end, the Chinese government has launched a series of policies and measures, which include, for example, the recently released statements of the Communist Party of China (CPC) Central Committee and State Council on the implementation of sustainable development and the realization of national carbon emission targets (SCPRC 2021; CPCCC and SCPRC 2021a).

Based on China’s national conditions, to achieve the carbon peak and carbon neutrality goals, the country should coordinate international and domestic energy resources, promote advanced green and low-carbon technologies and experience, pursue cooperation to cope with climate change, and constantly enhance international information exchange and communication. In addition, the relationship between pollution and CE reduction and energy security, industrial chains, supply chain security, food security, and people’s normal lives should be properly considered. The economic, financial, and social risks that may accompany green and low-carbon transformation should be effectively reduced, safety should be ensured, and CE reduction should be achieved.

Under the above principles, an economic system of green and low-carbon circular development will initially take shape by 2025, the energy utilization rate of industry as a whole will be greatly improved, the energy consumption associated with GDP will be reduced by 13.5% below the 2020 level, the CE stemming from GDP will be reduced by 18% below the 2020 levels, and the proportion of nonfossil energy consumption will reach approximately 20%. By 2030, China’s CE per unit of GDP will decrease by more than 65% below the 2005 levels, and the proportion of nonfossil energy consumption will reach approximately 25%. By 2060, the proportion of nonfossil energy will exceed 80% (DES 2021).

### Establishing a High-Carbon Sink Economic Model—Vigorous Development of a High-Carbon Absorption Economy

In existing studies, a high-CA economy is regarded as an integral part of a low-carbon economy, namely, these studies only emphasize partially offsetting CEs through carbon sequestration. Carbon is absorbed by ecosystems or carbon capture and storage (CCUS) technology is adopted, that is, CO_2_ is captured before emission, transported to storage locations through pipelines or ships, and finally compressed and injected underground to achieve the purpose of complete emission reduction. We have carried out a comparative analysis based on the Dujiangyan model of eliminating harm and promoting benefits, which was widely implemented in ancient China (Dujiangyan Government 2019). This model has been widely popularized in the field of natural hazard-related disaster prevention and control. We believe that construction of both high-CA and low-CE economic models must be treated similarly in order to obtain the same drive to reduce CEs, increase the number of carbon sinks, and fully manifest the high potential of biological system photosynthesis in CA. The potentially irreplaceable role of lower plant algae and other microorganisms in the high-CA process (carbon sequestration) is especially important. Our experimental research on the Qinghai-Tibetan Plateau revealed that the development of a high-light *Chlorella* algae cultivation system could improve the CA capacity by 15–20 times over the local natural ecosystem level (Yu et al. 2022), and high-light microalgae cultivation on the Qinghai-Tibetan Plateau could greatly improve the CA capacity in the region.

The Chinese government has noted that by 2025 forest coverage will reach 24.1% and the forest volume will reach 18 billion m^3^. By 2030, these indicators will reach 25.0% and 19 billion m^3^, respectively, thereby laying a solid foundation for realizing the goal of carbon neutrality, and creating a new realm of harmonious coexistence between humans and nature (SCPRC 2021; CPCCC and SCPRC 2021a). These documents further clarified and recommended measures for the CA of ecosystems, namely, land spatial planning and use control should be strengthened, the red line of ecological protection should be strictly followed, ecological space occupation should be closely controlled, and carbon sequestration in existing forestlands, grasslands, wetlands, oceans, soils, permafrost areas, karst regions, and so on should be stabilized. The scale of new construction land should be strictly controlled, and flexible utilization of urban and rural construction sites should be promoted. Land use standards should be effectively implemented, economical and intensive land use should be enhanced, land-saving technology and models should be promoted, and the capacity of CA in the land system should be improved (NPC and CPPC 2021).

### Establishing a Complementary Model of Traditional and Clean Energy—Systematic Optimization of the Regional Zero-Carbon Economy

Due to regional differences in natural and socioeconomic development conditions, there exist notable differences in energy structure at the global, national, and regional scales. For example, Shandong Province and Qinghai Province, which are both located in the Yellow River Basin in China, exhibit very different energy structures (CPCCC and SCPRC 2021b). In terms of CA and CE, the CEs in Shandong Province in 2019 (256 million tons of carbon) were 18.1 times higher than those in Qinghai Province (SPS 2021), while the CA reached only 8.12% of that in Qinghai Province. The ratio of the annual average CE to the CA in Shandong Province is 232.5, which means that it is a high-risk province with CEs far exceeding the provincial CA capacity. In contrast, the ratio for Qinghai Province reaches only 0.85, which indicates that it is a low-risk province with net negative emissions (QPS 2021). These two provinces could cooperate in the carbon trading market to alleviate the pressure of CE reduction in Shandong Province. In terms of the energy structure, in 2020 thermal power accounted for 88.4% of the total power generation above a scale of 578.1 billion kWh in Shandong Province, while clean energy accounted for 89.3% of the total power generation capacity of 94.8 billion kWh in Qinghai Province. At present, the clean energy power generation capacity in Qinghai Province reaches only 15.7% of the thermal power generation capacity in Shandong Province, and a large amount of external transmission and consumption is needed. Under the carbon peaking and carbon neutrality goals, Shandong Province will gradually reduce the power generation proportion and CEs originating from thermal power (mainly coal power) in the future. The energy demand after thermal power withdrawal can fully absorb the green power in Qinghai Province, and there remains enough room for improvement in green power consumption in the future.

How can regional CE differences attributed to energy structure differences be solved to achieve regional and national carbon neutrality and minimize the national CER? The Chinese government emphasized the necessity of green and low-carbon development planning in achieving its carbon peak and neutrality goals. In particular, the corresponding requirements of these goals should be fully incorporated into medium- and long-term social and economic development planning, therefore enhancing specific development planning at all administrative levels. Additionally, coordination among various plans is essential to ensure the implementation of these national carbon goals in the main development directions, social policies, and major projects undertaken across different scales. The spatial pattern of green and low-carbon development should be optimized by careful layout of major infrastructure and productivity components, public resources should be continuously enhanced, and a new pattern of land spatial development and protection should be established.

The green and low-carbon transition of local development guidance, tasks, and industries is critical for the success of major regional development strategies, such as the ecological protection and high-quality development of the Yellow River Basin and the Yangtze River Economic Belt. Achieving these goals requires not only accelerating the development of emerging industries, such as new-generation information technology, biotechnology, new energy, new materials, and high-end equipment, but also enhancing green and low-carbon industries by integrating advanced information technologies, such as the big data, artificial intelligence, and fifth-generation mobile communication.

Therefore, to stimulate in-depth cooperation and synergy between Qinghai and Shandong, for example, it is necessary to address clean energy consumption in Shandong, transfer high-energy consumption enterprises from Shandong to Qinghai in an orderly manner, and replace traditional energy in Shandong with clean energy in Qinghai to establish a complementary interregional energy system. It is also necessary to systematically optimize the water resource allocation mechanism in the upper, middle, and lower reaches of the Yellow River Basin as well as maximize the regional coordination mechanism for clean energy such as hydropower. Enhanced coordination and cooperation between industrial carbon reduction and regional exchange must increase to jointly realize a zero-carbon economy in the Yellow River Basin. All of these coordinated improvements must be supported by the ultrahigh-voltage (UHV) power grid in the Yellow River Basin and the establishment of a CE trading market.

## Conclusion

Carbon peaking and carbon neutrality are not only important strategic actions to cope with global climate change, but also they represent a new and important research topic related to global climate change. In the field of Earth system sciences, carbon peaking and neutralization have been accepted as important research frontiers. CEs, which are mainly based on human social and economic activities, are the main disaster-causing factor that initiates and increases environmental risks. Therefore, we have developed the CER system, including its technical terms, core content, and methodology, to enhance research on environmental risks. We have proposed the CER concept and defined the CER index. The CER is a dynamic indicator based on the balance between CE and CA.

This method was applied to estimate the risks in different provinces across China. The results show a spatial pattern of high risk in eastern China and low risk in western China, and there is no CER in the Qinghai-Tibetan Plateau. A closer examination of the Qinghai-Tibetan Plateau’s CER shows significant variation in the last two decades, which is mainly attributed to the trend of climate warming and more frequent extreme climatic events. Adaptation policies should consider this variation in order to establish and achieve local carbon peak and neutralization targets. We also propose a regional synergy system for potential CER governance. This system is a “two-wheel” drive mechanism with low CE and high CA. The government emphasizes that carbon emission reduction is the main priority and that increasing CA is a supplement. Our study demonstrates that to achieve China’s goals, increasing CA is equivalent to reducing CEs, and it is necessary to promote the “high carbon absorption economy” and to support the government in setting goals for CER governance.

We recognize the limitations of the CER. Our analysis largely consists of an analysis of CEs and CA. Various uncertainties could influence the magnitude of the outcomes highlighted here. The first concerns the warming scenarios and emissions pathways. A higher warming scenario, for example, 2.0 °C above preindustrial levels, may lead to smaller transition effects than a 1.5 °C warming scenario, given the lower degree of emission reduction and deviation from today’s production and consumption patterns that it entails.

A second uncertainty concerns carbon emission accounting. Although the types of emission activities covered by the existing CE inventory are very comprehensive, the classification is not sufficiently detailed, and there is no unified inventory for the accounting of CEs at different regional scales. This makes the composition of CO_2_ emission accounting somewhat ambiguous. The current accounting method for CE identificaion is based on the CEs of industrial energy consumption. But CO_2_ emissions are not related only to the industry and transport sectors. With the advent of the new urbanization and post industrialization era, the energy consumption of the residential sector accounts for approximately 13% of the total energy consumption of the whole society (NBS 2020). This has become the main source of CO_2_ emissions (Jiang, Xue, Ma, et al. 2020). Due to a lack of micro data, for example, on household energy consumption, proposing and implementing a scientific CE accounting inventory based on regional characteristics is still an important challenge in research and policy formulation.

The third uncertainty relates to the terrestrial carbon budget. Although the CA function of terrestrial ecosystems has been confirmed by the academic community, the current understanding of the size, distribution, dynamics, and driving factors of terrestrial CA is still largely limited. This uncertainty involves many uncertain aspects, including differences in the definitions of terrestrial CA proposed by different researchers, sampling errors caused by an uneven distribution of sample points, and differences in model processes and parameters. At the same time, there are also large differences between the CA intensities obtained by different methods. In particular, due to the uncertainty in the lateral flux measurement, combining the bottom-up method and the top-down method still remains a challenge.

We recommend that future research addresses the following four topics: (1) How can the CEs of the whole life cycle of regional activities be measured? (2) How can regional CA be calculated based on different climate and socioeconomic development scenarios? (3) How can CER vulnerability be mitigated from the macrolevel to the microlevel? (4) How can a regional synergy model be obtained through the “two-wheel drive” of CE reduction and CA?
